# Efficiency and selectivity of cost-effective Zn-MOF for dye removal, kinetic and thermodynamic approach

**DOI:** 10.1007/s11356-023-25919-4

**Published:** 2023-02-27

**Authors:** Abeer S. Elsherbiny, Ahmed Rady, Reda M. Abdelhameed, Ali H. Gemeay

**Affiliations:** 1 Chemistry Department, Faculty of Science, Tanta University, Tanta, 31527 Egypt; 2 Applied Organic Chemistry Department, Chemical Industries Research Institute, National Research Centre, Dokki, Giza 12622 Egypt

**Keywords:** Metal-Organic frameworks, Waste batteries, Green synthesis, Dye adsorption

## Abstract

Green synthesis of metal–organic frameworks (MOFs) has attracted a lot of attention as a crucial step for practical industrial applications. In this work, green synthesis of zinc(II) metal–organic framework (Zn-MOF) has been carried out at room temperature. The Zn metal (node) was extracted from spent domestic batteries, and the linker was benzene di-carboxylic acid (BDC). The characterization of the as-prepared Zn-MOF was accomplished by PXRD, FT-IR spectroscopy, SEM, TEM, TGA, and nitrogen adsorption at 77 K. All the characterization techniques strongly supported that as-synthesized Zn-MOF using metallic solid waste Zn is similar to that was reported in the literature. The as-prepared Zn-MOF was stable in water for 24 h without any changes in its functional groups and framework. The prepared Zn-MOF was tested for the adsorption of three dyes, two anionic dyes, aniline blue (AB), and orange II (O(II)) as well as methylene blue (MB), an example of cationic dye from aqueous solution. AB has the highest equilibrium adsorbed amount, *q*_*e*_, of value 55.34 mg g^−1^ at pH = 7 and 25 °C within 40 min. Investigation of the adsorption kinetics indicated that these adsorption processes could be described as a pseudo-second-order kinetic model. Furthermore, the adsorption process of the three dyes was described well by the Freundlich isotherm model. According to the thermodynamic parameters, the adsorption of AB on the prepared Zn-MOF was an endothermic and spontaneous process. In contrast, it was non-spontaneous and exothermic for the uptake of O(II) and MB. This study complements the business case development model of “solid waste to value-added MOFs.”

**Supplementary Information:**

The online version contains supplementary material available at 10.1007/s11356-023-25919-4.

## Introduction

The circular economy was launched in 2010, as well as several principles resembling recyclability, waste valorization, renewability, and sustainability (Kaur et al. 2018; Payne et al. 2019). When these concepts are put into practice, a new economy emerges that focuses on greening the life cycle of items from production to disposal. If a smart, integrated circulation system is not implemented for the management of environmental challenges, there will be a significant hazard to the environment. Utilizing waste to create useful materials for a wide range of applications will maximize waste management’s contribution to green property operations. Various waste supplies are known, resembling agriculture wastes, plastic, vegetable, fruit, biological, electronic, and petroleum refinery wastes (Periyasamy et al. 2020). The generation of electronic waste (e-wastes) has become a significant problem, especially in developing countries. Spent lithium ions and domestic alkaline batteries form a sizable fraction of e-wastes (Deep et al. 2016). Recovering spent alkaline batteries could provide significant economic and environmental advantages. Hence, if recycling technologies are utilized properly, around 20,000 tons of metallic elements is expected to be fixed each year instead of through landfilling or incineration (Gallegos et al. 2013). Zinc or manganese products that have been recovered can be employed in a variety of industrial applications (Gallegos et al. 2013).

Another issue that threats the environment is the pollution of organic dyes. According to the World Health Organization (WHO), environmental contaminants found in the soil, air, and water cause 25% of human health diseases (Naresh et al. 2018). Among these pollutants, organic dyes are currently widely utilized in plastics, cosmetics, textiles, paper, and prescribed drug industries (Gemeay et al. 2020, Al-Zawahreh, Barral, et al. 2021, Beydaghdari et al. 2022a). However, organic dyes cause serious air, water, and soil contamination, end in fatal environmental pollution, and are a health threat (Wang et al. 2012, Johnson et al. 2014, Chen et al. 2015, Han et al. 2015, Wu et al. 2016, He et al. 2018, Paiman et al. 2020, Beydaghdari et al. 2022a, b). Therefore, sewer water ought to be effectively treated before discharge into the natural environment. So far, varied approaches for the removal of dyes from wastewater are reported such as ion exchange, advanced oxidation processes (AOPs), biological processes, coagulation processes, membrane separation, and adsorption processes. (Zhou et al. 2010; Zou et al. 2012; He et al. 2015; Du et al. 2022; Shi et al. 2022; Tang et al. 2023). Due to its low cost, effectiveness, and simplicity, selective adsorption of organic dyes has become vital for protecting both human health and the environment (Zou et al. 2012; Azimi et al. 2017; Roy and Stoddart 2021; Jrad et al. 2022). The finding of new adsorbents or modifications of existing adsorbents for achieving higher adsorption capacity and better adsorption conditions has always been of interest (Kadhom et al. 2020). Metal–organic frameworks (MOFs) have been introduced as high-performance adsorbents for dye removal applications (Abbasi et al. 2020; Xie et al. 2020; Beydaghdari et al. 2022a; Singh et al. 2022b). This is attributed to their high surface area, porosity, chemical stability, and structural flexibility (Akpomie and Conradie 2020; Khan et al. 2020; Wu et al. 2021; Singh et al. 2022b). Therefore, authors are operating during this area of interest to build up MOF adsorbents to get rid of organic dyes from wastewater. MOFs are a sub-category of coordination polymers with unique merits (Diercks et al. 2018). They are a promising class of crystalline porous hybrid materials composed of metal ion clusters linked by organic linkers via strong covalent bonds to form extended networks (Yaghi and Li 1995; Kitagawa et al. 2004; Férey 2008; Hamoud et al. 2022).

MOFs present a variety of engineering properties like large porosity, high surface area, potentially high density of active sites, and acceptable thermal and chemical stability (Beydaghdari et al. 2022a; Singh et al. 2022b). MOFs have applications for photocatalysis (Du et al. 2021; Hamoud et al. 2022; Liu et al. 2022a, b), storage (Ben et al. 2012, Li et al. 2018, Li et al. 2019, Jia et al. 2022), and sensing (Lustig et al. 2017; Yan et al. 2021). Recently, some authors have reported the removal of cationic and anionic dyes using different types of MOFs (Cook et al. 2013; Yang et al. 2018; Liu et al. 2020; Paiman et al. 2020; Xie et al. 2020; Raza et al. 2021; Beydaghdari et al. 2022a). Scientists must look into alternative application fields and find ways to lower MOF synthesis costs to prepare MOFs for possible commercialization. In this study, Zn metal extracted from waste batteries was recovered and used to make a highly valuable product appealing to industry and falls under the category of MOFs. The prepared Zn-MOF contains Zn metal extracted from the spent Zn batteries and commercial terephthalic acid as a linker. The physicochemical properties of Zn(BDC)-MOF were studied using X-ray diffraction (XRD), Fourier transform infrared (FT-IR), scanning electron microscopy–energy-dispersive X-ray spectroscopy (SEM–EDX), nitrogen adsorption at 77 K, and transmission electron microscope (TEM). The structure of the as-prepared Zn-MOF was compared to the commercial one mentioned in the literature. Moreover, the efficiency and the selectivity of the prepared Zn-MOF towards the removal of three dyes, namely, aniline blue (AB), acid orange II (O(II)), and methylene blue (MB) from aqueous solutions were investigated.

## Experimental

### Materials

**Fig. 1:**
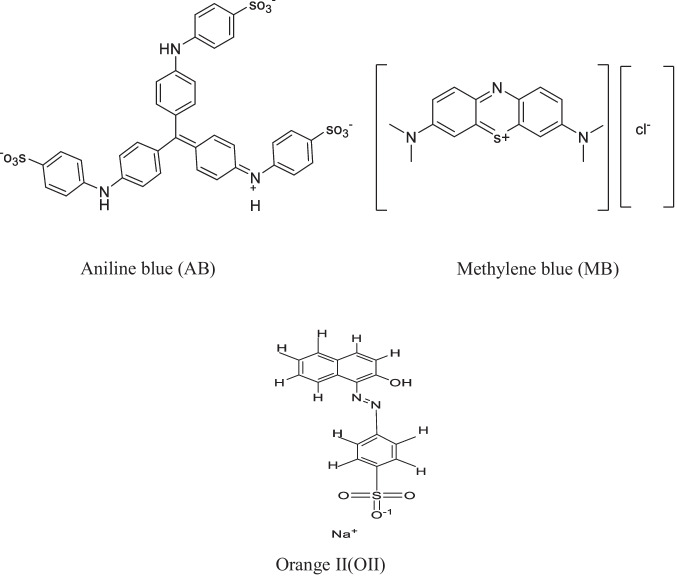
Chemical structures of dyes

Zn metal was collected from energizer battery strips. Benzene dicarboxylic acid (BDC) was obtained from LANXESS, Belgium. *N*,*N*-dimethylformamide (DMF), triethylamine (TEA), and chloroform were purchased from LANXESS, Belgium. Nitric acid and ethyl alcohol were obtained from El Nasr Pharmaceutical Chemical, Egypt. Aniline blue (AB), methylene blue (MB), and orange II dye (O(II)) (Fig. 1) were received from Sigma-Aldrich. All the reagents and solvents were analytical grade and used as received. The adsorption experiments were done utilizing doubly distilled water.

#### Synthesis of Zn(BDC)-MOF

Zn(BDC)-MOF was prepared at room temperature as the following: waste battery strips (1 g) were dissolved in 6 mL of concentrated HNO_3_ then diluted up to 100 mL with distilled water to produce Zn(NO_3_)_2_, “solution 1.” Benzene dicarboxylic acid (H_2_BDC) (5 g) was dissolved in 50 mL DMF with a stirring, “solution 2.” The two solutions were mixed which give rise to a white precipitate. Then, an extra volume of DMF was added dropwise with stirring until the white precipitate disappears. Triethylamine (3 mL) was added dropwise to the mixture with stirring. After stirring for 24 h, a white product is filtered off and washed with 30–40 mL of DMF. The precipitate was immersed in chloroform for 24 h to extract the DMF. The obtained precipitate was dried in an oven at 120 °C for 24 h and then activated at 100 °C under a vacuum to yield the evacuated product. The yield of Zn(BDC)-MOF was about 1.9 g at the end.

#### Characterization

The prepared Zn-MOF was characterized for its structure and morphology using several techniques. Fourier transform infrared spectroscopy (FT-IR) was recorded using Shimadzu FT-IR-8101A spectrophotometer within the range (4000–400 cm^−1^) using the potassium bromide disc technique. Powdered X-ray diffraction (PXRD) was performed on a Philips PW1710 instrument equipped with Cu Kα radiation (*λ* = 1.5418 Å). The sample was subjected to the range of 2θ = 5° to 70° with a scan rate of 8 (° min^−1^) at room temperature. Nitrogen adsorption/desorption measurements at 77.35 K were carried out using NOVA-touch (Quantachrome Instrument, USA) to determine the pore structure of Zn(BDC) material. Before the measurements, the sample was degassed at 100 °C for 2 h. The surface area of Zn(BDC) was determined using the Brunauer–Emmett–Teller (BET) equation. The shape and microstructure were examined by scanning electron microscopy. High-resolution TEM (HRTEM) is a potent method for observing local structures. Thermogravimetric analysis (TGA) was recorded on Themys One^+^ (SETARAM) under air flow of 25 mL min^−1^. The samples (5–10 mg) were heated from room temperature to 800 °C at a linear heating rate of 20 °C min^−1^. The value of the surface zeta potential was recorded on zeta potential analyzer (NICOMP, 380 ZLF, USA). UV–Vis spectrophotometer UV-1650 (Shimadzu) (200–800 nm) was used to track down changing the adsorbed amount of the dyes on Zn-MOF. The prepared Zn-MOF was dried in an oven under a vacuum (Suszarka Prozniowa-SPR). A bench pH meter (AD1030, Adwa, Hungary) was used to adjust the pH of the medium.

#### Adsorption experiments

Batch adsorption experiments were carried out as the following: 30 mg of Zn (BDC) was mixed with 10 mL of [MB] = 12 mg L^−1^ and [O(II)] = 35.03 mg L^−1^ dyes, meanwhile 10 mg of Zn (BDC) was mixed with 10 mL of [AB] = 59.02 mg L^−1^ dye in a stoppered Erlenmeyer flask. The suspension was then shaken for 120 min on a shaker water thermostat (110 rpm) at 25 °C. Thereafter, the suspension was separated by centrifugation (model LCEN-402N), and the absorbance of the filtrate was recorded on Shimadzu UV–Vis spectroscopy at *λ*_max_ = 595, 485, and 660 nm for AB, O(II), and MB, respectively. The adsorption capacity at a time (*t*), (*q*_*t*_), and at equilibrium (*q*_*e*_) of the three dyes were estimated by Eqs. 1 and 2, respectively

1 $${q}_{t}=\left({C}_{0}-{C}_{t}\right) V/m$$

2 $${q}_{e}=\left({C}_{0}-{C}_{e}\right) V/m$$

where *C*_0_, *C*_*e*_, and *C*_*t*_ are the initial, equilibrium concentrations and at a time (*t*) (mg L^−1^) of three dyes, respectively. *V* is the volume (L) of the solution, and *m* is the mass (g) of Zn(BDC). The mean values of each assay were reported after being carried out in duplicate for each. To optimize the factors affecting the adsorption process, various parameters were investigated. The effect of pH (2.0–11.0) using universal buffer, temperature (293–313 K), contact time (3–120 min), and the initial concentration of dyes was studied using the one-factor-at-a-time technique by changing the studied factor while holding the others constant.

#### Adsorption isotherms and kinetics

Several isotherm models such as the Langmuir (Eq. 3), Freundlich (Eq. 4), Temkin (Eq. 5), and Dubinin–Radushkevich (D–R) (Eq. 6) were applied to the adsorption experimental data to study the interaction of Zn(BDC) with the three dyes.

(Langmuir 1916):

3 $${C}_{e}/{q}_{e}=\left(1/{q}_{max}{K}_{L}\right)+{C}_{e}/{q}_{max}$$

(Freundlich 1906):

4 $$\mathrm{ln}{q}_{e}=\mathrm{ln}{K}_{F}+\left(1/n\right)\mathrm{ln}{C}_{e}$$

(Dubinin and Radushkevich 1947):

5 $$\mathrm{ln}{q}_{e}=\mathrm{ln}{q}_{max}-B {\varepsilon }^{2}$$

(Temkin and Pyzhev 1940):

6 $${q}_{e}={B}_{1}\mathrm{ln}{K}_{T}+{B}_{1}\mathrm{ln}{C}_{e}$$

where *K*_*L*_ is the Langmuir constant associated with the adsorption energy (L g^−1^), *q*_max_ is the maximum amount of dye adsorbed to form a monolayer (mg g^−1^), *K*_*F*_ is the Freundlich constant which shows roughly the adsorption capacity, 1/*n* is the intensity of adsorption, *ε* is the Polanyi potential that equals to RT ln (1 + 1 / *C*_*e*_), *B* is a constant related to the mean free energy of (mg^2^ J^−2^), *B*_1_ is constant equals to RT / *b*, and *K*_*T*_ is the Temkin constant.

Three distinct kinetic adsorption models consisting of pseudo-first-order (PFO), pseudo-second-order (PSO), and intraparticle diffusion (ID) were applied (Lagergren 1898, Weber Jr and Morris 1963; Elsherbiny et al. 2017). The linear rate relationships of these kinetic models are given in Eqs. 7 and 8, respectively

7 $$\mathrm{log}\left({q}_{e}-{q}_{t}\right)={\mathrm{log}q}_{e}-\left({k}_{1}/2.303\right)t$$

8 $$t/{q}_{t}=1/{k}_{2}{q}_{e}^{2}+t/{q}_{e}$$

9 $${q}_{t}={k}_{p} {t}^{{~}^{1}\!\left/ \!{~}_{2}\right.}+C$$

where *k*_1_ and *k*_2_ are the first- and second-order rate constant of adsorption, respectively. *k*_*p*_ is the rate constant of the intraparticle diffusion (mol g^−1^ min^0.5^), and *C* is a constant (mg g^−1^) presenting data about the boundary thickness of the layer. The bigger *C* value, the more influence the boundary layer.

#### Reusability of Zn(BDC)-MOF

Cyclic adsorption–desorption tests were performed to evaluate the reusability of Zn(BDC). In this experiment, NaOH (1 M) and ethanol were used as recovery to regenerate Zn(BDC). After the first cycle, the collected amount of loaded Zn(BDC) (0.15 g) was shaken with 10 mL of NaOH solution for 60 min in the case of AB, while ethanol in the case of O(II) and MB, respectively. The recovered adsorbent was washed with water followed by ethanol after that DMF, then chloroform was dried in an oven at 120 °C for 24 h, and then activated at 100 °C under a vacuum. The obtained recovered Zn(BDC) was reused again for another cycle of adsorption of each dye. Five adsorption–desorption rounds were carried out to examine the potential reusability of the as-prepared Zn(BDC).

## Results and discussion

### Characterization

#### FT-IR spectra

**Fig. 2:**
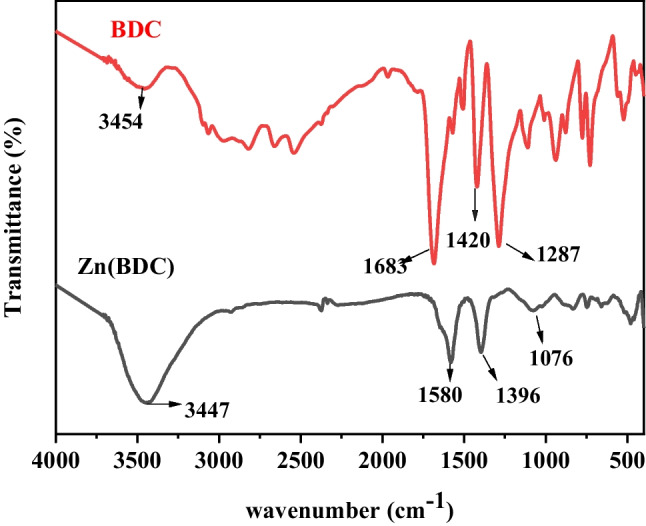
FT-IR spectrum of as-synthesized Zn(BDC) and commercial BDC

The obtained Zn(BDC)-MOF was well characterized by several analytical and spectroscopic techniques. Figure 2 shows FT-IR spectra of commercial benzene dicarboxylic acid and the prepared Zn(BDC)-MOF. Different peaks appeared in the spectra of the prepared Zn(BDC) such as the strong signal at 3447 cm^−1^ assigned to OH of carboxylic acid or traces of adsorbed water. The strong peaks at 1580 cm^−1^ and 1396 cm^−1^ are due to the asymmetric and symmetric stretching vibration of –COO^−^, respectively (Wu et al. 2013). The peaks at 821 cm^−1^ and 746 cm^−1^ indicate the aromatic C–H out-of-plane bending (Wu et al. 2013; Wang et al. 2014). No peaks of protonated H_2_BDC (1715–1680 cm^−1^) were observed, which indicates that the organic linker (BDC) is bonded with the metal center to form Zn-MOF (Akbarzadeh et al. 2020).

## Powdered XRD

**Fig. 3:**
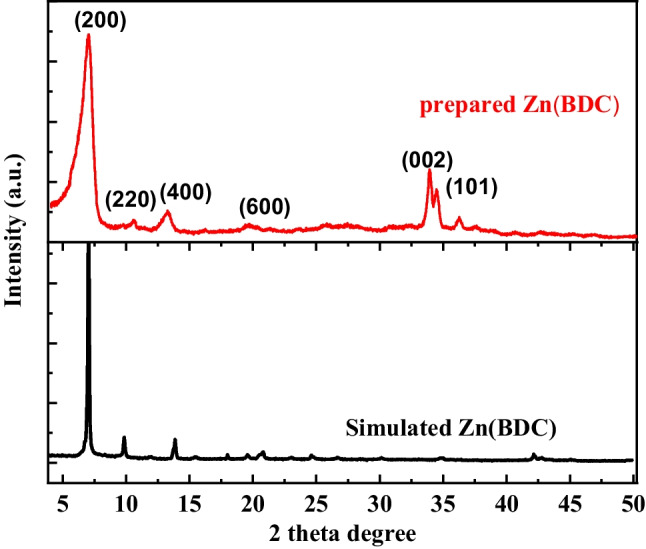
XRD pattern of as-synthesized Zn(BDC) compared with the simulated one

Significant information can be obtained by using the PXRD technique, such as crystal structure, phase type, and product purity. PXRD pattern for the waste-prepared Zn(BDC) is shown in Fig. 3. As revealed in this figure, the diffraction pattern of waste Zn(BDC) was matched well with that of simulated Zn(BDC) as reported in the literature (Jasmina Hafizovic et al. 2007; Getachew et al. 2014; Bakhtiari and Azizian 2015; Wang et al. 2017; Ruan et al. 2018; Niu et al. 2021; Sharma et al. 2021). The presence of major diffraction peaks of Zn(BDC) at 7.07°, 10.57°, and 13.27° which are assigned to the lattice planes of (200), (220), and (400), respectively (Dang et al. 2020). The appearance of these characteristic peaks suggested the successful preparation of Zn(BDC). The diffraction peaks at 34.01° and 36.27° are related to *d*_002_ and *d*_101_ of ZnO nanoparticles (Arefi and Rezaei-Zarchi 2012). Furthermore, sharp peaks in this pattern confirm the crystalline structure of waste Zn(BDC). By using of Debye–Scherrer equation (Eq. 10) (Singh et al. 2011), the average particle size of the prepared Zn(DBC) was calculated, and it was about 29.03 nm which confirm the nanostructure of the prepared waste Zn(BDC).

10 $$D=0.94\lambda /\beta \mathrm{cos}\theta$$

## Brunauer–Emmett–Teller measurement

**Fig. 4:**
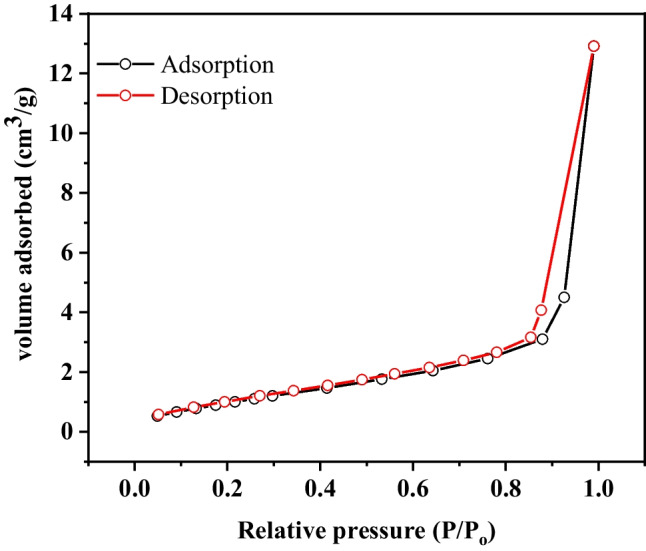
N_2_ adsorption–desorption isotherm for Zn(BDC)

The specific surface area and the pore size distribution of prepared Zn(BDC) were achieved from the nitrogen adsorption–desorption isotherms at 77 K. The nitrogen isotherm of the obtained Zn(BDC)-MOF exhibited a type I isotherm indicating a microporous structure (Fig. 4) (Dang et al. 2020). The pore size distribution was calculated through the DFT method resulting in an average pore total pore volume of 9.76 nm and 0.47 cm^3^ g^−1^, respectively. The large pore volume of prepared Zn(BDC) makes it a good adsorbent for environmental remediation. Additionally, the Brunauer–Emmett–Teller (BET) surface area was measured and equals 96.28 m^2^ g.^−1^

## TGA measurement

**Fig. 5:**
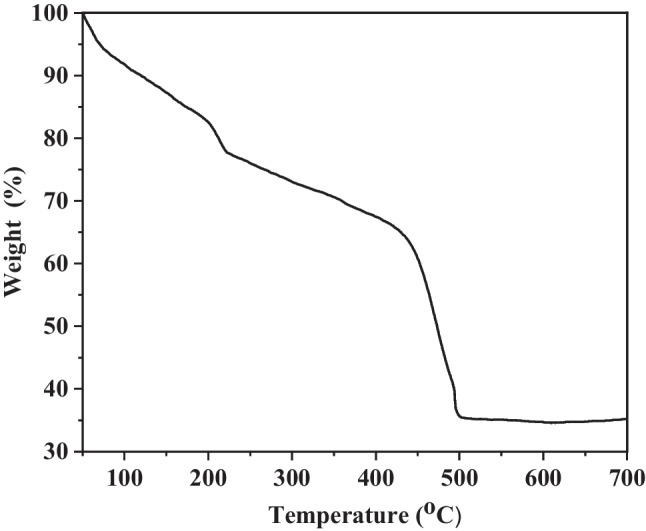
TGA of prepared Zn(BDC)

The thermal stability of the synthesized Zn(BDC) was investigated by thermogravimetric analysis (TGA). The TGA curve of Zn(BDC) exhibits a low weight loss at a temperature below 100 °C due to the loss of solvent molecules (Fig. 5). The decomposition of organic linkers occurred in the range of 200–220 °C with weight loss of about 22% (Rallapalli et al. 2010; Dang et al. 2020). The thermogram indicates that Zn(BDC) is thermally stable up to 425 °C, and its framework collapses at 425 °C. With further heating, the weight loss percentage reaches a constant at 65.4% above 500 °C (Rani et al. 2020). The results suggest that the considerably high stability of the synthesized Zn(BDC) toward temperature could be an advantage for the removal of pollutants at high temperatures.

## SEM and TEM images

**Fig. 6:**
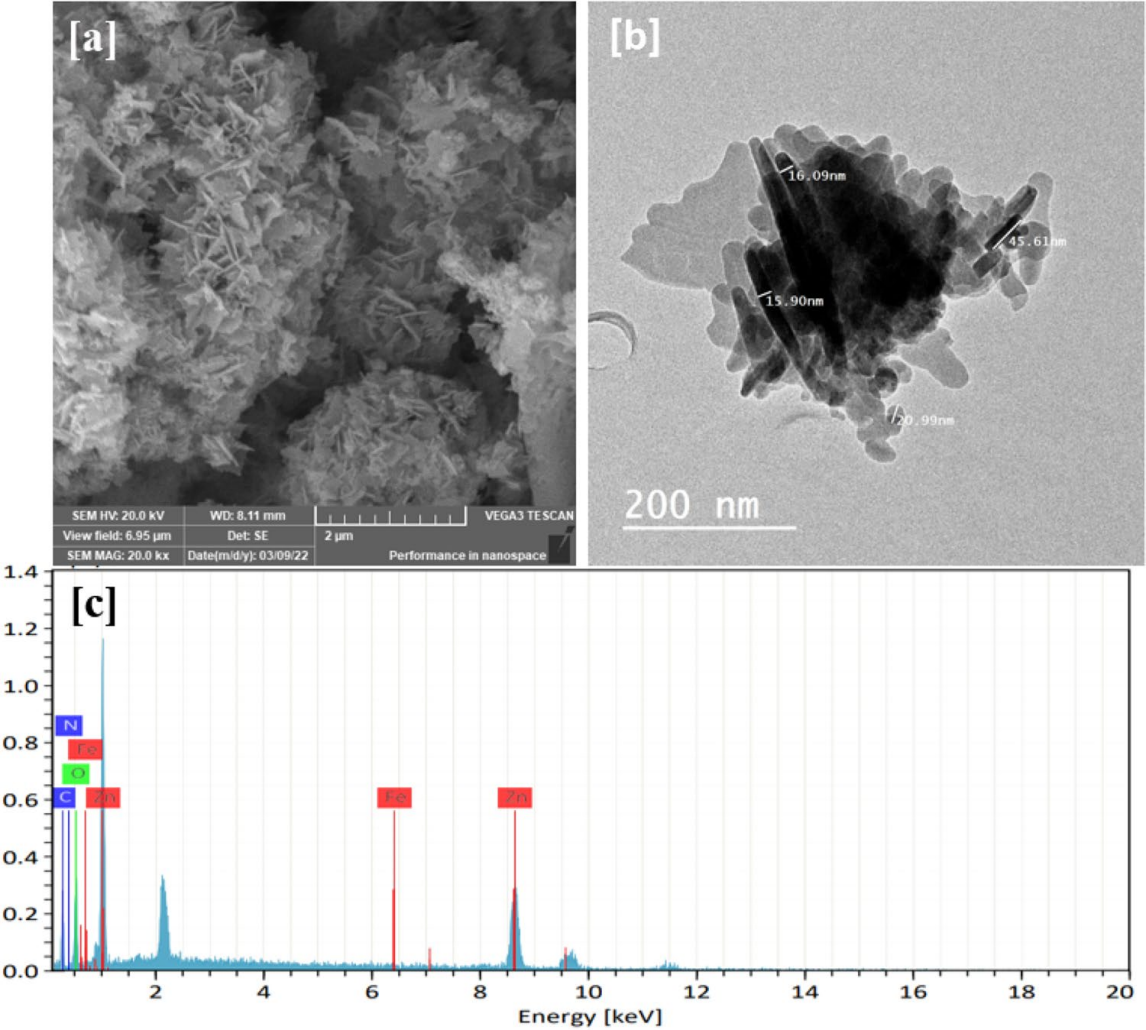
**a** SEM, **b** TEM image, and **c** EDX of as-synthesized Zn(BDC)

The morphology of MOF materials was successfully analyzed using scanning electron microscopy. Figure 6a displays the SEM image of Zn(BDC) which has rod-like crystals. Also, the crystals agglomerated together on the surface forming large spheres. This agglomeration increases the overall particle size of Zn(BDC). Furthermore, the TEM image (Fig. 6b) confirmed the rod-like shape with an average length of 159.3 nm and a width of 14.53 nm. These values approved the nanostructure of the prepared Zn(BDC) as was evidenced by XRD patterns. The purity and elemental composition of the synthesized Zn(BDC) were determined using energy-dispersive X-ray (EDX) spectroscopy. The composition of Zn(BDC) was established to have carbon, nitrogen, oxygen, and zinc with ratios of 49.10, 0.16, 29.56, and 21.06, respectively, as shown in Fig. 6c. Finally, the total characterization using XRD, FT-IR, EDX, and morphology analysis confirms the successful synthesis of Zn(BDC) using Zn metal extracted from waste batteries.

## Water and mechanical stability

**Fig. 7:**
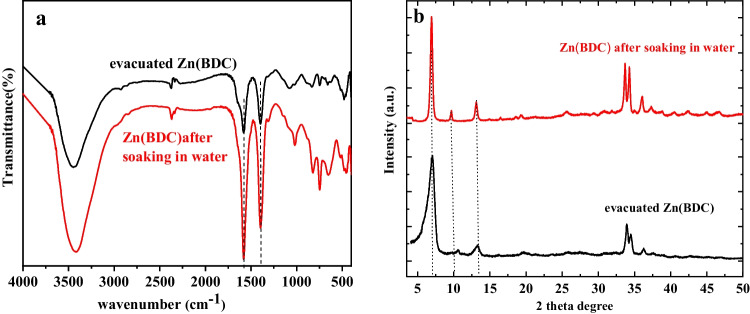
**a** FT-IR spectra and **b** XRD of evacuated as well as water suspended Zn(BDC)

Adsorption removal of the dyes was performed in aqueous solutions with shaking; therefore, a water stability test was done by comparing FT-IR and PXRD of the evacuated dry and water-suspended Zn(BDC) samples. A certain amount of Zn(BDC) was suspended in an amount of water and shacked for 24 h, and the FT-IR and PXRD of the separated Zn(BDC) were measured. Water had no observable impact on the functional groups of Zn(BDC) as seen in Fig. 7a. The PXRD of evacuated and soaked Zn(BDC) samples appeared in Fig. 7b. The main diffraction peaks of Zn(BDC) were 7.07°, 10.57°, and 13.27° that had no significant change indicating no structure degradation of Zn(BDC) and keeping their crystallinity even after 24 h in suspension. Since the adsorption equilibrium of the dyes from the water was typically accomplished within 24 h (Alqadami et al. 2018; Nanthamathee and Dechatiwongse 2021), it was concluded that the prepared Zn(BDC) is highly stable and convenient for the adsorption process.

## Zeta potential and point-zero charge measurements

To study the surface charge properties of prepared Zn(BDC), the zeta potential and the pH of point of zero charges (pHpzc) were determined. The surface of Zn(BDC) has a positive charge with an average value of 35.34 mV, and the (pHpzc) value equals 7 in water as shown in Fig. S1a and b. The value of zeta potential points out a stable colloidal solution of Zn(BDC) is formed in water which is suitable for the adsorption of anionic dyes on its surface (Dai 1994).

## Adsorption isotherm

The efficacy of the synthesized Zn(BDC)-MOF in the removal of dyes from an aqueous solution was investigated. Three dyes namely (MB), an example of cationic dye, AB, and O(II) as examples of anionic dyes were selected. Different parameters, including pH, contact time, adsorbent dosages, and dye concentration, were assessed at a constant stirring rate to delve deeper into the adsorption behavior. Meanwhile, all experiments were accomplished three times.

## Impact of pH

**Fig. 8:**
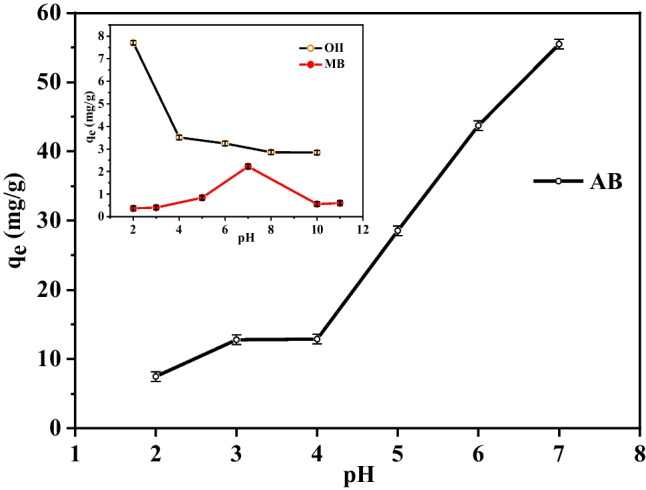
The effect of initial solution pH on dyes adsorption onto Zn(BDC); [AB] = 59.02 mg L^−1^, [O(II)] = 35.03 mg L^−1^, [MB] = 12 mg L^−1^, wt. of Zn(BDC) = 0.01 g and 0.03 g for AB, O(II) and MB, respectively at 25 °C

The pH of the solution affects the surface charge of the materials and dye molecules in the solution (Dai et al. 2018). Therefore, it significantly has an important role in the interaction and adsorption process. Additionally, the presence of several functional groups on the surfaces of dyes and MOF, which are typically protonated/deprotonated in response to pH changes, encourages electrostatic contact between the two phases (Raza et al. 2021). The effect of pH on the adsorption of the three dyes on Zn(BDC) was studied using a universal buffer in the 2–11 range except for AB. It has been reported that AB dye could be decolorized under an alkaline environment (Rashtbari et al. 2020); therefore, its experiments were done only in acidic and neutral mediums. As shown in Fig. 8, the values of the equilibrium adsorbed amount, *q*_*e*_, were 55.5 mg g^−1^, 3 mg g^−1^, and 2.22 mg g^−1^ for AB, O(II), and MB, respectively, at pH 7. It was observed that AB dye has the highest adsorbed amount on Zn(BDC). However, there are a significant changes in the adsorbed amount of O(II) and MB dyes. In addition, the minimum adsorption of dyes observed at high pH could be attributed to the competition between the anionic dye molecules with the OH^−^ ions. It was reported that the adsorption of dyes on MOF’s surface is assigned to the electrostatic interactions between the dye molecules and the polar sites of MOF. Moreover, the non-covalent interactions such as hydrogen bonding and π–π interactions along with the aromatic ring in the MOF structure have a significant role in the adsorption phenomenon (Muhambihai et al. 2020). The highest adsorbed amount of AB on Zn(BDC) may be due to the existence of the three negative sulphonate groups in AB which strongly interact with a positive charge on Zn(BDC) surface at lower pH values. Also, the non-bonding electrons (*n*) of these sulphonate groups have been considered to be strong *n*-electron donors (Chen et al. 2008). As a result, the *n*–π and π–π electron-donor–acceptor (EDA) interactions could be formed between sulphonate, and aromatic groups of AB and benzene ring of Zn((BDC) result in adsorption on the various MOF crystal faces (Muhambihai et al. 2020). The adsorbed amount of AB was descended by decreasing the pH from 7 to 2. This phenomenon is due to the protonation of sulphonate groups of AB and subsequently declines its interaction with the positively charged Zn(BDC) surface. However, the maximum adsorbed amount of O(II) was obtained at pH equal to 2 which may be explained by the H-bond between the N = N group in its structure and protonated active sites of Zn(BDC) surface. The higher adsorbed amount of AB than O(II) recommends that the electrostatic interaction and EDA are the more efficient forces responsible for the adsorption on Zn(BDC)-MOF. The lowest adsorbed amount of cationic MB may be attributed to the π–π interaction along with the aromatic rings in the MOF structure.

## Effect of contact time

**Fig. 9:**
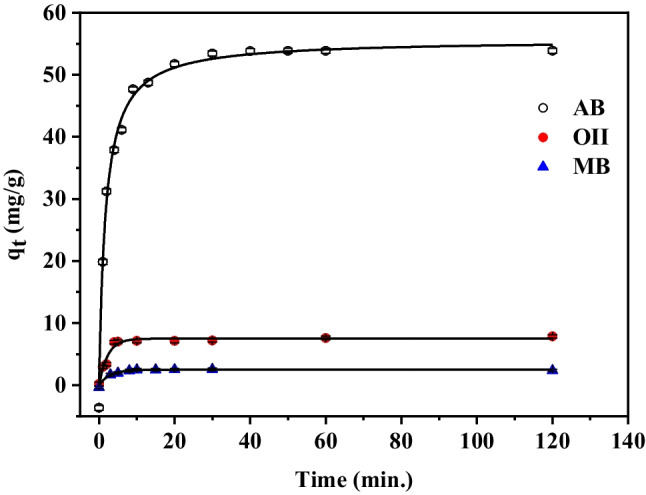
The effect of contact time on the adsorption of dyes on Zn (BDC); [AB] = 59.02 mg L^−1^, [O(II)] = 35.03 mg L^−1^, [MB] = 12 mg L^−1^, wt. of Zn(BDC) = 0.01 g and 0.03 g for AB, O(II) and MB, respectively at 25 °C

The influence of contact time on the adsorbed amount of AB, O(II), and MB on Zn(BDC) was studied under pH = 7 (except O(II) at pH = 2) and at different contact times from 5 to 120 min as displayed in Fig. 9. The uptake of three dyes on Zn(BDC) was rapid in the beginning of contact time with different values of the adsorbed amount. After that, a slight increase in the adsorbed amount till the equilibrium was maintained. AB was the most rapidly adsorbed within the first 12 min with *q*_*e*_ of 48.74 mg g^−1^ and reached the equilibrium after 40 min with *q*_*e*_ = 54.84 mg g^−1^. Small amounts of O(II) and MB adsorbed on Zn(BDC) with *q*_*e*_ = 7.86 mg g^−1^ and 2.63 mg g^−1^, respectively at 10 min. The successful capture of AB by Zn(BDC) could be ascribed to the existence of interaction sites (three negative sulphonate groups) in AB molecular structure. The initial rapid dye uptake on the MOFs may have been caused by the high dye concentration gradient force quickly filling the material’s pores and active sites (Rashtbari et al. 2020). The pores and adsorption sites eventually reached saturation, achieving equilibrium (Jiang et al. 2016; Wang et al. 2018, Singh et al. 2022a). Moreover, the results showed that the removal behavior of organic dye was specific and closely related to both dye structure and MOF properties (Wang et al. 2018). Besides, the maximum adsorption capacity of the three dyes on Zn(BDC) was compared with other potent adsorbents as listed in Table 1. The synthesized waste Zn(BDC) MOF displayed high uptake capacity for AB in neutral medium compared with the other surfaces which worked in acidic medium.

**Table 1:** The comparison of the maximum adsorption capacity for the adsorption of three dyes onto various adsorbents reported in literature

Dye	Adsorbent	*q*_max_ (mg g^−1^)	Conditions	References
AB	Zn(BDC)-MOF Dead biomass of Aspergillus fumigatus Pomegranate peel	55.34 11.05 19.51	0.01 g/10 mL, *C*_0_ = 59.13 mg L^−1^, pH = 7, 298 K for 60 min 50 mg L^−1^ of dye solution, 1 g L^−1^ of sorbent and 60 min 0.1 g, pH = 4, 303 K	This study Baskar et al. (2014) Usman, Aftab et al. (2021b, a)
MB	Zn(BDC)-MOF Co-doped Fe(BDC)-MOF Raw mango seed Fe_3_O_4_@ZIF-8	2.632 23.916 25.36 20.2	0.03 g/10 mL, *C*_0_ = 53.188 mg L^−1^, pH = 7, 298 K for 120 min 0.01 g/10 mL, for 24 h 1 g/100 mL, *C*_0_ = 100 mg L^−1^, time = 60 min at 300 K (10 mg) was added to dye with shaking at room temperature for 15 h	This study Soni et al. (2020) Senthil Kumar et al. (2014) Zheng et al. (2014)
OII	Zn(BDC)-MOF MMT MIM-MMT BIM-MMT GO PRGO	10.01 1.26 2.29 1.70 8.4 32.5	0.03 g/10 mL, *C*_0_ = 12.823 mg g^−1^, pH = 2, 298 K for 120 min *C*_0_ = 10 mg L^−1^ for 24 h at pH = 2 2 g/3 mL, *C*_0_ = 66.7–300 mg L^−1^ for 24 h at 298 K	This study Yılmazoğlu et al. (2022) Zhang et al. (2019) Zhang et al. (2019)

## Adsorption kinetics

**Fig. 10:**
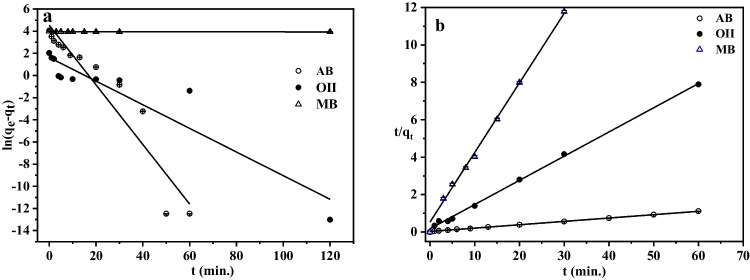
Linear plot of **a** pseudo-first-order and **b** pseudo-second-order kinetics model for AB, O(II), and MB adsorbed on Zn(BDC)

To obtain relevant information about the rate of sorption as well as the adsorption mechanism concerned in the process, the kinetics of adsorption of AB, O(II), and MB onto Zn(BDC) (in Fig. 9) was analyzed using PFO and PSO models. The linear plots of the two models are presented in Fig. 10a, b. The calculated parameter values and their correlation coefficient (*R*^2^) are listed in Table 2. Kinetic studies revealed that the linear regression (*R*^2^) of PSO model was comparatively higher than that of PFO model and close to unity for the three dyes. Additionally, an extremely small variation between the experimental adsorption capacity (*q*_e,exp_) values and the PSO theoretical value (*q*_e,cal_) supports that the PSO model was a robust model with high accuracy (Solgi et al. 2017). Another research work reported a similar observation (Azhdari et al. 2019). The diffusion mechanism of the three dyes on the Zn(BDC) was clarified from the IDM illustrated in Fig. S2. The regression of *q*_*t*_ versus *t*^1/2^ was linear for three diffusion steps with zero intercept value. These steps are film diffusion, intraparticle diffusion, and adsorptive attachment intersected regions. The third stage is very slow, and it is the rate-limiting step. This recommended that there were additional rate-controlling processes besides intraparticle diffusion. The external mass transfer had a significant impact on the adsorption process as well (Ma et al. 2012; Jiang et al. 2018; Samuel et al. 2018; Abdulhameed et al. 2019). Therefore, external mass transfer and intraparticle diffusion worked together to govern the overall adsorption process (Ma et al. 2012).

**Table 2:** Kinetic data with their correlation coefficient (*R*^2^) for the adsorption of dyes on Zn(BDC)

Model	Parameters	Dyes		
		AB	O(II)	MB
Pseudo-first order	*q*_*e*,exp_ (mg g^−1^)	53.84	7.857	2.546
	*q*_*e*,cal_ (mg g^−1^)	93.17	5.093	1.006
	*K*_1_ (min^−1^)	0.2691	0.1067	0.183
	*R*^2^	0.9073	0.8797	0.7996
Pseudo-second order	*q*_*e*,cal_ (mg g^−1^)	55.34	7.899	2.689
	*K*_2_ (min^−1^)	0.0127	0.0870	0.2598
	*R*^2^	0.9990	0.9993	0.9980
Intraparticle diffusion	*Kp*_1_(mg g^−1^ min^−0.5^)	14.14	3.157	0.9813
	R^2^	0.9239	0.9317	0.9765
	*Kp*_2_ (mg g^−1^ min^−0.5^)	4.104	0.1155	0.0095
	*R*^2^	0.8984	0.6995	0.7389
	Kp_3_(mg g^−1^ min^−0.5^)	0.5970	0.0550	0.0012
	*R*^2^	0.9990	0.9488	0.9999

## Effect adsorbent amount

The working capacity is one of the practically important adsorbent performance indicators. So, the effect of the amount of Zn(BDC)-MOF on the adsorption of the three dyes was studied (Fig. S3). The amount of Zn(BDC) was varied in the 0.005–0.04 g range in the case of AB and O(II) dyes, which was changed in the 0.02–0.1 g range for MB dye at pH = 6 and at 25 °C. The *q*_*e*_ values of the three dyes were increased continuously with increasing the amount of Zn(BDC). There was no further increment in *q*_*e*_ of AB at a higher amount of Zn(BDC) than 0.015 g. However, an increment in the amount of Zn(BDC) more than 0.03 and 0.1 g does not show any significant change in the uptake of O(II) and MB, respectively. The adsorption efficiency increased with an increase in Zn(BDC) due to the availability of more adsorption sites and consequently a larger surface area for dye uptake. Similar results were mentioned in the literature (Sharma et al. 2021; Soltani et al. 2021; Usman, Aftab, et al. 2021b, a). Moreover, the smallest amount of Zn(BDC) which is saturated with AB refracted the highest selectivity of the surface towards AB.

## Effect of dye concentration and isotherm studies

The effect of initial dye concentration was investigated in the range of 14–147 mg L^−1^, 7–87 mg L^−1^, and 3–32 mg L^−1^ for AB, O(II), and MB, respectively. The optimal experimental conditions were established. As appeared in Fig. S4, increasing the initial concentration of the three dyes had a positive effect on the values of *q*_*e*_. This is because a high concentration allows more effective interaction with the MOFs’ active sites, which in turn leads to more dye being fixed to the surface and optimal uses of the adsorptive sites (Rehman et al. 2019). These are consistent with the results of the abstraction of anionic and cationic dyes from wastewater onto the natural magnetic adsorbent (Sanad et al. 2021). Again, AB dye has the highest adsorption ability on Zn(BDC) which is confirmed by the above results. Four isotherm models were applied by using the data presented in Fig. S4. The calculated parameters obtained from plotting Eqs. 3–6 and their correlation coefficient (*R*^2^) are stated in Table 3. The results revealed the adsorption data of the three dyes did not obey Langmuir model due to the low correlation coefficient (< 0.2) of the linear plots. However, the results in Table 3 showed that the Freundlich model is suitable to describe the adsorption process of three dyes on Zn(BDC) (Niu et al. 2021). The applicability of the Freundlich model indicated that the adsorption process mainly occurred on a heterogeneous surface with multilayer adsorption (Tanhaei et al. 2015; Habiba et al. 2017). The intensity factor (*n*) was more than unity in the case of AB and increased with temperature, suggesting that AB is more amenable to adsorption onto Zn(BDC) at higher temperatures (Elsherbiny et al. 2012). The favorability of the adsorption process at higher temperature was confirmed by increasing the value of *K*_*F*_ as the temperature was raised (Aljeboree et al. 2017). In contrast, the intensity factor has a value less than unity, and the value of *K*_*F*_ decreased as the temperature raised for O(II) and MB, indicating the adsorption process was unfavorable which is confirmed by the small value of *q*_*e*_ for both dyes. However, the adsorption of MB has (*n*) value close to unity at 298 K and this value declined with increasing the temperature, confirming that the adsorption process was unfavorable at a higher temperature. Furthermore, the *R*^2^ value of Temkin model showed the availability of this model to fit the adsorption data of the three dyes. For AB, the value of *K*_*T*_ increased with increasing the temperature reveals the positive effect of temperature on the binding energy of AB with Zn(BDC) surface. In contrast, the temperature harms *K*_*T*_ value of O(II) and MB*.*

**Table 3:** Isotherm parameters and the correlation coefficient (*R*^2^) for the adsorption of dyes onto Zn(BDC)

Model	Parameters	293 K			303 K			313 K		
		AB	O(II)	MB	AB	O(II)	MB	AB	O(II)	MB
Freundlich	*n*	1.041	0.245	0.983	1.050	0.252	0.908	1.211	0.211	0.782
	*K*_*F*_ × 10^−2^ (L mg g^−1^)	777.6	0.044	52.10	812.0	0.022	28.98	845.2	0.0008	23.53
	*R*^2^	0.9583	0.951	0.974	0.943	0.9704	0.9751	0.9355	0.9926	0.9534
D–R	*q*_*m*_ (mg g^−1^)	89.06	84.66	4.015	107.7	77.94	3.395	117.8	70.74	1.389
	*K*_D–R_ × 10^−6^ (mol^2^ k J^−2^)	− 2.985	− 50.11	− 1.689	− 2.939	− 69.39	− 2.778	− 2.770	− 114.9	− 3.279
	*E* (kJ mol^−1^)	0.4093	0.0989	0.5441	0.4124	0.0849	0.4243	0.4248	0.0660	0.3905
	*R*^2^	0.7125	0.9739	0.8828	0.7545	0.9940	0.9273	0.7998	0.9991	0.8602
Temkin	*B*_*T*_ (J mol^−1^)	66.56	39.68	2.406	85.38	34.55	2.134	84.72	33.81	2.604
	*K*_*T*_ (L mg^−1^)	0.3529	0.1149	0.6527	0.3680	0.0939	0.4818	0.3919	0.0723	0.4362
	*B*	36.60	61.33	1012	29.51	72.91	1180	30.72	76.97	999.3
	*R*^2^	0.9421	0.9886	0.9330	0.9591	0.9947	0.9456	0.9725	0.9671	0.9519

The D–R model’s mean adsorption energy (*E*) value (Ma et al. 2012) can be utilized to distinguish between chemical and physical adsorption. If *E* < 8 kJ mol^−1^ physisorption dominates, if *E* is 8–16 kJ mol^−1^, the mechanism is either chemisorption or ion exchange, and if *E* > 16 kJ mol^−1^, it implies the existence of only chemisorption. (Ploychompoo et al. 2021). The poor *R*^2^ values of D–R model in Table 3 revealed that this model was not suitable to fit the experimental adsorption data of AB and MB on Zn(BDC). But it is appropriate to depict the adsorption of O(II) with *E* value less than 8 kJ mol^−1^ implying that the process was dominated by physical adsorption. Additionally, its value declines as the temperature rose, confirming the process is favorable at lower temperatures.

## Effect of coexisting ions

Real wastewater may contain several cationic or anionic salts which may influence the uptake of dye pollutants by the adsorbent. Thus, to check the influence of such coexisting cations and anions, batch adsorption experiments were carried out in the presence of various ions such as K^+^, Na^+^, Ca^2+^, NO_3_^−^, SO_4_^2−^, and Cl^−^. In the experiment, 50 mg L^−1^ of these ions was mixed with each dye by a 1:1 ratio keeping the other optimal experimental conditions established. The results revealed that the removal efficiency of AB did not affect anymore the presence of these ions in the solution. This reflects the higher selectivity of Zn(BDC) towards AB dye. The removal efficiency of O(II) was slightly affected by the presence of these ions in water. It was declined from 67.29 to 61.03%, 62.73%, 64.64%, 62.73%, 61.73%, and 42.38% in the presence of Na^+^, K^+^, and Ca^2+^, Cl^−^, NO_3_^−^, and SO_4_^2−^, respectively. However, the removal efficiency of MB was strongly affected by the presence of these ions in water. It decreased from 63.67 to 14.91%, 12.54%, 18.18%, 12.45%, 19.51%, and 21.98%; removal efficiency of O(II) was slightly affected by the presence of these ions in water. This is attributed to the lower selectivity of Zn(BDC) towards MB and the competition of these ions with MB to adsorb on the surface of Zn(BDC). The same phenomenon was observed by several authors (Hua et al. 2018; Patra et al. 2020; Shahnaz et al. 2020).

## Thermodynamic investigations

With the aid of the Van’t Hoff equation, the effect of temperature in the range of 293–313 K was investigated to understand the thermodynamic parameters of the adsorption process of AB, O(II), and MB on Zn(BDC). It was noticed that temperature had a positive effect on the adsorbed amount of AB on Zn(BDC), while it has a negative effect for O(II) and MB. This indicates the endothermic nature of uptake of AB and the exothermic for O(II) and MB, respectively, as listed in Table 4. The positive value of Δ*H*° for AB adsorption on Zn(BDC) confirms that the process is endothermic, which links an increase in the temperature to increase the adsorption of AB. This is comparable to earlier studies on the sorption of dye on MOFs (Gautam et al. 2017, Arora et al. 2019; Nanthamathee and Dechatiwongse 2021). Moreover, the small positive value of Δ*H*° (< 40 kJ mol^−1^) of AB indicates that the adsorption process was monitored by physical adsorption (Konicki et al. 2017). However, the negative value of ΔH^°^ for the uptake of O(II) and MB reflects the exothermic nature of the process. Similar results were found for the adsorption of MB on Cu(BDC) (Doan et al. 2020). The other thermodynamic parameters were calculated using Eqs. (2) and (3) in supporting information. and their values are recorded in Table 4. The negative Δ*G*° for the adsorption of AB on Zn(BDC) was found at all temperatures, and its value increases with an increase in temperature, implying that the adsorption process was spontaneous and more efficient at higher temperatures. The low Δ*G*° values (< 20 kJ mol^−1^) suggest that the adsorption process was physisorption in nature. The adsorption of both O(II) and MB dyes on Zn(BDC) that have positive Δ*G*° implies that the process was non-spontaneous. The highest positive value of Δ*G*° was observed at 313 K for both dyes. The positive Δ*S*° value for adsorbed AB showed that the disorder increased during the adsorption process (Yang et al. 2017). For the adsorbed O(II) and MB, Δ*S*° had negative values revealing that the randomness at the solid–liquid interface was decreased (Yu et al. 2017; Zhang et al. 2020). A similar trend was noted for the removal of MB using Cu(BDC) (Doan et al. 2020). The smaller Δ*S*° value for the adsorption of MB on Zn(BDC) than O(II) indicates the process was more ordered.

**Table 4:** Thermodynamic parameters of the three dyes adsorbed onto Zn(BDC)

Temp (°K)	*K*_*d*_ (L g^−1^)			∆*H*° (kJ mol^−1)^			∆*G*° (kJ mol^−1^)			∆*S*° (J mol^−1^ K^−1^)		
	AB	O(II)	MB	AB	O(II)	MB	AB	O(II)	MB	AB	O(II)	MB
293	7.860	0.7592	0.5773	9.196	− 29.22	− 25.27	− 5.060	0.610	1.447	48.66	− 101.8	− 91.18
303	9.322	0.5515	0.3574				− 5.547	1.628	2.359			
313	10.00	0.3522	0.2984				− 6.033	2.646	3.271			

## Regeneration and reusability

**Fig. 11:**
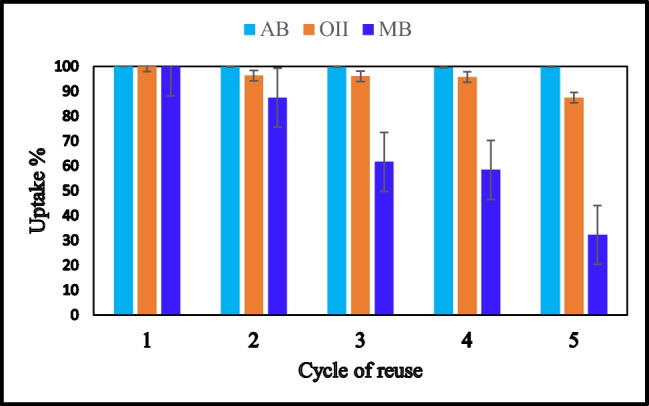
Reusability of Zn(BDC) for the adsorption of three dyes

**Fig. 12:**
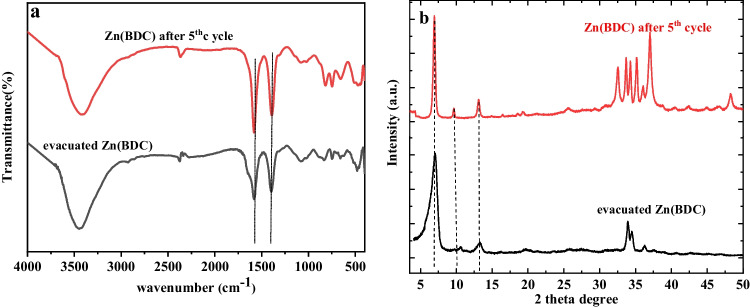
**a** FT-IR spectra and **b** XRD of evacuated Zn(BDC) as well as Zn(BDC) after 5th cycle

One of the most crucial points for applicable adsorbent is its ability for regeneration and reuse. Moreover, the employed desorbing agent for the regeneration process should be inexpensive and non-destructive to the adsorbent. To test the reusability of Zn(BDC), it was subjected to successive adsorption–desorption cycles using 0.1 M NaOH as the desorbing agent for adsorbed AB and ethanol for O(II) and MB. Five cycles of adsorption–desorption were performed and are presented in Fig. 11. The results exhibited a good desorption and recovery performance for AB dye with an adsorption efficiency of 99.7% after 5 cycles. However, for O(II), a reasonable decrease in its uptake reached 87.4% in the fifth cycle, while MB showed a great decrease in its uptake by Zn(BDC) after 5 cycles. The reusability results confirm the ability of the prepared Zn-MOF to remove the anionic dyes more than the cationic ones. Moreover, the FT-IR and PXRD of the regenerated Zn(BDC) after five adsorption–desorption cycles were conducted (Fig. 12a, b). FT-IR measurements showed no change in the functional groups of Zn(BDC) after the fifth cycle of regeneration. PXRD presented no observable change in the crystalline structure of Zn(BDC). The diffraction peaks at 32.58°, 33.69°, 34.28°, 35.21°, and 37.01° are due to ZnO which released after fifth cycle. However, Zn(BDC) still hold its framework and crystallinity, since the main diffraction peaks 7.07°, 10.57°, and 13.27° appeared.

## Conclusion

In this work, Zn(BDC)-MOF was successfully prepared from waste batteries as a source of Zn ion with the green method. The structure of the as-prepared Zn(BDC)-MOF coincided with commercially prepared one as confirmed by FT-IR and XRD. The obtained Zn-MOF was applied to remove two anionic dyes (AB and O(II)) and a cationic dye (MB). The prepared Zn-MOF was more efficient for the removal of anionic dyes than the cationic one. The maximum adsorption capacities of AB, O(II), and MB were found to be 55.34 mg g^−1^, 10.01 mg g^−1^, and 2.63 mg g^−1^, respectively. The adsorption of AB, O(II), and MB was well fitted by the Freundlich isotherm model, while the adsorption kinetic data followed the pseudo-second-order model. The enthalpy change (∆*H*°) and Gibbs free energy change (∆*G*°) showed that the adsorption process was spontaneous and endothermic processes for AB, however, non-spontaneous and exothermic for the uptake of O(II) and MB. Moreover, the prepared Zn(BDC)-MOF can be easily regenerated and reused for the removal of AB for five cycles without a decrease in the adsorption capacity. Zn(BDC)-MOF showed good stability after five cycles of adsorption–desorption with no changes in their functional groups and framework.

### Supplementary Information

Below is the link to the electronic supplementary material.

Supplementary file1 (DOCX 247 KB)

## Data Availability

All the data and materials are available in the manuscript.
