# Supplementary material for: Efficiency and selectivity of cost-effective Zn-MOF for dye removal, kinetic and thermodynamic approach
Source: Environ Sci Pollut Res Int. 2023 Feb 27;30(49):106860–75. doi: 10.1007/s11356-023-25919-4 (PMC10611857; doi:10.1007/s11356-023-25919-4)
Supplement: Supplementary file 1 — Supplementary file1 (DOCX 247 KB) [file 11356_2023_25919_MOESM1_ESM.docx]

# Efficiency and selectivity of cost-effective Zn-MOF for dyes removal, kinetic and thermodynamic approach

Abeer S. Elsherbiny^1^, Ahmed R. Algad^1^, Reda M. Abdelhameed^2^, Ali H. Gemeay^1^

*^1^Department of Chemistry, Faculty of Science, Tanta University, Tanta 31527, Egypt*

*^2^Applied Organic Chemistry Department, Chemical Industries Research Institute, National Research Centre, Dokki, Giza, 12622, Egypt*


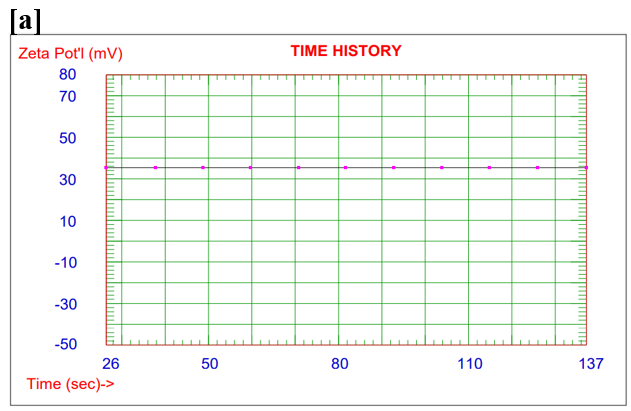


Fig. S1. (a) Zeta potential, (b) Zero point charge of Zn(BDC).

Fig. S2. Intraparticle diffusion plot of adsorption of dyes on Zn(BDC)

Fig. S3. Effect of adsorbent dosage on adsorption of dyes on Zn(BDC).

Fig. S4. Effect of initial concentration on adsorption of dyes on Zn(BDC).

The parameters of adsorption thermodynamics can be calculated by introducing the experimental data at three different temperatures into the following Equations.

$$\ln K_{d}= -\frac{\Delta H_{\mathrm{ads}}}{\mathrm{RT}}+\frac{{\Delta S}_{\mathrm{ads}}}{R} (1)$$

$$\Delta G_{\mathrm{ads}}= -RT\ln K_{d} \left( 2 \right)$$

$$\Delta G_{\mathrm{ads}}=\Delta H_{\mathrm{ads}}-T\Delta S_{\mathrm{ads}} \left( 3 \right)$$

Where; K_d_ is the distribution coefficient (K_d_ = q_e_/C_e_), ΔG_ads_ is the change in Gibbs-free energy of adsorption process, $\Delta H_{\mathrm{ads}}$ (kJ.mol^-1^) is the enthalpy change, and $\Delta S_{\mathrm{ads}}$ (J.mol^-1^.K^-1^) is the entropy change.
